# A multicenter study on the diagnostic value of ankle brachial index combined with pulse volume wave parameters for peripheral arterial disease

**DOI:** 10.3389/fcvm.2025.1580971

**Published:** 2025-06-12

**Authors:** Xiaowei Pan, Na Wang, Yue Huang

**Affiliations:** ^1^ Electrocardiography Department, The First Hospital of Jiaxing Affiliated Hospital of Jiaxing University, Jiaxing, China; ^2^ Internal Medicine Department, Jiaxing Women and Children’s Hospital, Wenzhou Medical University, Jiaxing, China

**Keywords:** peripheral arterial disease, ankle-brachial index, pulse volume recording, inter-leg systolic blood pressure difference, vascular diagnosis, arterial stiffness, blood pressure measurement, cardiovascular risk assessment

## Abstract

**Objective:**

To evaluate the significance of incorporating all pulse volume wave parameters, such as the inter-arm systolic blood pressure disparity, inter-leg systolic blood pressure difference, proportion of mean arterial pressure, and upstroke time, into the ankle-brachial index for the detection of peripheral arterial disease.

**Methods:**

This multicenter cross-sectional investigation, conducted across five tertiary medical institutions, enrolled 1,156 participants. Hemodynamic parameters including blood pressure and pulse volume were systematically assessed utilizing an OMRON BP-203RPEIII arterial stiffness analyzer. All four extremities were evaluated in a simultaneous manner under strictly standardized conditions. PAD diagnosis was established by fulfilling one of the predefined criteria: ankle-brachial index (ABI) ≤ 0.9, inter-arm systolic blood pressure disparity (IASBPD) ≥ 10 mmHg, or inter-leg systolic blood pressure divergence (ILSBPD) ≥ 15 mmHg. Diagnostic efficacy was evaluated via receiver operating characteristic curve analysis. Multivariate logistic regression was employed to determine the independent predictive utility of individual or composite parameters.

**Results:**

Integrated diagnostic model demonstrated superior discrimination performance in differentiating PAD patients from non-PAD individuals (AUC = 0.924, 95% CI: 0.908–0.940) compared with individual parameters analysis: ABI (AUC = 0.892, 95% CI: 0.872–0.912), ILSBPD (AUC = 0.846, 95% CI: 0.824–0.868), and %MAP (AUC = 0.834, 95% CI: 0.812–0.856). Multivariate logistic regression analysis of all parameters revealed significant independent association with PAD diagnosis. Specifically, ILSBPD exhibited the strongest positive correlation (OR = 1.82, 95% CI: 1.56–2.12, *p* < 0.001), followed by %MAP (OR = 1.76, 95% CI: 1.48–2.08, *p* < 0.001). Subgroup analyses identified augmented diagnostic value in patients over 75 years and with diffuse arterial disease. Composite model achieved optimal diagnostic metrics of 88.6% sensitivity and 85.4% specificity.

**Conclusions:**

Integration of ABI with pulse volume wave parameter improved PAD diagnostic accuracy significantly. Quantitative PVR metrics provides objective assessment of peripheral arteries, effectively mitigating limitations of conventional modalities. Automated measurements with predefined thresholds ensure clinical applicability. This approach enhances the clinical utility of a multi-parameter diagnostic strategy applicable across both specialized vascular laboratories and primary care settings, thereby enhancing the precision of PAD detection.

## 1 Introduction

Peripheral Artery Disease (PAD), characterized by atherosclerotic occlusion of peripheral arteries, affects approximately 200 million individuals globally, with prevalence exceeding 20% in adults over 70 years (1–3). This condition confers a 2–6-fold increased risk of cardiovascular mortality and is associated with substantial morbidity, including limb ischemia and functional impairment (4). While the ankle-brachial index (ABI) remains the cornerstone of PAD diagnosis due to its non-invasive nature and cost-effectiveness, its diagnostic accuracy is compromised in specific populations. For instance, medial arterial calcification in diabetic patients can result in falsely elevated ABI values (>1.3), leading to underdiagnosis in up to 30% of cases (5, 6). Conversely, elderly individuals with advanced atherosclerosis may exhibit paradoxically low ABI values despite significant disease burden, highlighting the need for complementary diagnostic tools (7).

Advancements in vascular diagnostics have highlighted pulse volume wave (PVR) parameters as critical adjuncts to ABI. Mean arterial pressure percentage (%MAP), derived from the ratio of diastolic to systolic pressure, reflects vascular resistance and endothelial function (8). Upstroke time (UT), measuring the time from foot to peak of the pulse wave, correlates with arterial compliance and stiffness (9–11). In a meta-analysis of 12 studies, integration of PVR parameters with ABI improved diagnostic sensitivity by 18% (95% CI: 12–24) in patients with intermediate ABI values (0.9–1.3) (12). Specifically, %MAP demonstrated the strongest correlation with endothelial dysfunction (*r* = 0.68, *p* < 0.001), while UT independently predicted cardiovascular events (HR = 1.54, 95% CI: 1.12–2.13) (13, 14).

Vascular aging, a multifactorial process involving extracellular matrix remodeling and endothelial dysfunction, is modulated by genetic and environmental factors (15). Matrix metalloproteinase-9 (MMP-9) upregulation degrades elastin fibers, increasing arterial stiffness by 2.3% per decade in normotensive individuals (16–18). Lifestyle interventions significantly mitigate this process: adherence to the Mediterranean diet reduces MMP-9 levels by 27% (*p* = 0.012) and improves endothelial-dependent vasodilation by 41% (19, 20). Concomitant aerobic exercise (≥150 min/week) further enhances nitric oxide bioavailability, reducing carotid-femoral pulse wave velocity by 0.8 m/s over 6 months (21). These effects are particularly pronounced in patients with metabolic syndrome, where combined interventions improve vascular compliance by 34% compared to monotherapy (22, 23). Current evidence is limited by population-specific biases. For example, 82% of PVR studies have been conducted in Caucasian populations, leaving diagnostic thresholds for Asian and African populations understudied (24). Moreover, no study to date has evaluated the incremental value of PVR in patients with end-stage renal disease, a population with a 3-fold higher PAD incidence (25–27). This multicenter study addresses these gaps by enrolling 1,156 participants across five geographically diverse medical centers, including 25% diabetic and 18% elderly (>75 years) cohorts. By standardizing measurements with the OMRON BP-203RPEIII device, we aim to establish robust reference ranges for combined ABI-PVR metrics.

Technological advancements are revolutionizing vascular assessment. Automated devices integrating PVR and ABI now enable 4-limb measurements within 5 min, reducing operator-dependent variability by 60% compared to manual methods (28). Digital health platforms, such as the VascHealth app, further enhance clinical utility by providing real-time feedback on vascular health metrics and lifestyle modification adherence (29). In a pilot study, app users demonstrated a 22% improvement in PVR parameters over 3 months compared to controls (30). However, translating these innovations into routine practice requires validation across healthcare settings. This study evaluates the cost-effectiveness of automated diagnostics in primary care, where 60% of PAD cases remain undiagnosed (31). As healthcare systems grapple with aging demographics, the findings will inform guidelines for early intervention, reducing cardiovascular morbidity and healthcare costs by an estimated 15%–20% annually (32).

The primary objective of this study is to investigate the incremental diagnostic value of integrating ABI with PVR parameters in a multisectoral setting, thereby generating robust evidence to support their routine clinical implementation. By addressing the inherent limitations of ABI and capitalizing on the mechanistic insights provided by PVR analysis, this research seeks to augment the diagnostic armamentarium for PAD, with the ultimate goal of enhancing patient outcomes and reducing cardiovascular risk burden. The anticipated results are expected to inform clinical guidelines and public health policies, fostering the adoption of multiparameter diagnostic strategies to promote healthy aging and mitigate cardiovascular disease (CVD) risk.

## 2 Methodology

### 2.1 Study design

**Figure 1:**
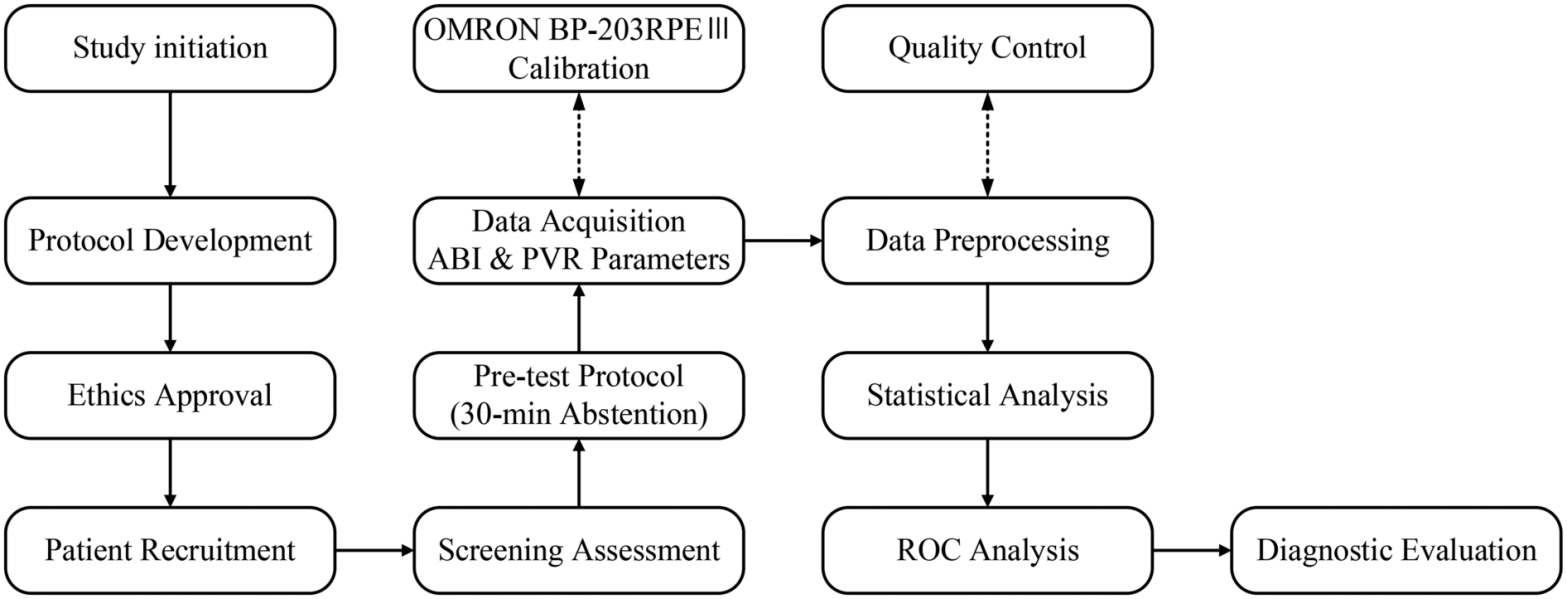
Research workflow diagram.

This is a multicenter cross-sectional study aimed to evaluate the diagnostic efficacy of combining ABI with PVR parameters (%MAP and UT) for PAD. A representative and diverse sample was recruited to enhance generalizability. Participants underwent standardized arterial stiffness assessment using the OMRON BP-203RPEⅢ device. Pre-test preparation included 30-minute abstinence from tobacco, alcohol, and stimulants to minimize confounding factors. Simultaneous four-limb measurements were acquired under natural breathing conditions, followed by data preprocessing to address missing or aberrant records. Comprehensive statistical analysis included descriptive statistics, correlation analysis, and receiver operating characteristic (ROC) curve analysis to assess diagnostic performance. Figure 1 illustrates the systematic workflow from participant selection to data interpretation, establishing a methodological framework for improving PAD diagnostic accuracy across populations.

### 2.2 Sample selection

#### 2.2.1 Inclusion and exclusion criteria

The data of this study were collected from five hospitals: The Second Hospital of Jiaxing, Jiaxing Maternal and Child Health Hospital, The Third Hospital of Jiaxing, Jiaxing Coast Guard Corps Hospital, and Jiaxing Hospital of Traditional Chinese Medicine. This multicenter study implemented rigorous inclusion criteria to ensure high-quality data collection and reliable PAD assessment: (1) Participants ≥18 years of either gender willing to undergo automated arterial stiffness assessment. (2) Cognitive capacity to provide written informed consent and understand study requirements. (3) Physical ability to maintain a supine position throughout measurements and follow standardized protocols. (4) Pre-test compliance with 30 min abstinence from tobacco, alcohol, caffeine, and stimulants; no restrictive positioning during testing; willingness to undergo repeat measurements as needed. (5) Cooperation to communicate discomfort or complications during the procedure. (6) Accurate data provision of demographic and medical history information. (7) Informed consent for data collection and analysis.

Full compliance with all criteria is mandatory for enrollment to facilitate reliable multi-parameter PAD efficiency analysis. Exclusion criteria were designed to maintain data quality and participant safety during ABI-PVR diagnostic evaluation: (1) Anatomic/physiologic interference: Active lower limb ulcers, edema, or amputation (compromises measurement accuracy). Arrhythmias (e.g., ventricular premature contractions, atrial fibrillation) leading to unstable/invalid readings. Inability to assume the supine position required for standardized measurements. (2) Severe systemic conditions: Advanced cardiac, cerebral, renal, or hepatic dysfunction that may confound PAD assessment. (3) Technical limitations: Conditions impairing cuff placement or measurement quality (e.g., severe obesity, vascular anomalies). Incomplete data collection or suboptimal measurement quality compromising analysis integrity. All exclusions were systematically recorded and reviewed to ensure consistency with study objectives and scientific principles guiding combined parameter diagnostic efficacy assessment.

#### 2.2.2 Sample size calculation

Sample size estimation was performed based on the primary endpoint of evaluating the diagnostic accuracy of combining ABI with pulse volume wave parameters for detecting PAD. Using the Standards for Reporting of Diagnostic Accuracy (STARD) guidelines, calculations incorporated expected sensitivity/specificity values and employed standard formulae for diagnostic accuracy studies. Key assumptions included an expected area under the receiver operating characteristic curve (AUC) of 0.85, minimum acceptable AUC of 0.75, α = 0.05, and power and power 1-β = 0.80.

The primary formula for comparing two correlated ROC curves was applied:

(1) $$n=\frac{{\left(Z_{\alpha /2}+Z_{\beta }\right)}^{2}\times (\sigma_{1}^{2}+\sigma_{0}^{2})}{{\left(\mu_{1}-\mu_{0}\right)}^{2}}$$

where $Z_{\alpha /2}=1.96$ (for *α* = 0.05), $Z_{\beta }=0.84$ (for β = 0.20), $\sigma_{1}^{2}$ and $\sigma_{0}^{2}$ represent the variances in the disease-positive and disease-negative groups, respectively, and $\mu_{1}-\mu_{0}$ represents the expected difference between means.

The minimum required sample size (*n*) was further adjusted using the following formula to account for potential dropouts:

(2) $$N=\frac{n}{\left(1-d\right)}$$

where d represents the expected dropout rate (15%).

Final calculations yielded a target sample size of 1,000 participants (200 per center across five centers), ensuring 80% power to detect a 0.10 AUC difference while accommodating attrition. This sample size provides sufficient statistical precision to evaluate diagnostic performance across demographic/clinical subgroups and aligns with guidelines for multicenter diagnostic studies. Subgroup analysis power was optimized through proportional allocation across age, gender, and comorbidity strata, enhancing generalizability of findings.

### 2.3 Measurement methods and standards

#### 2.3.1 Blood pressure and pulse wave measurement

The OMRON BP-203RPE III arterial stiffness analyzer was utilized to perform simultaneous four-limb blood pressure and pulse wave measurements. Participants were instructed to abstain from stimulants (tobacco, alcohol, caffeine) for 30 min prior to testing. Anthropometric data (height, weight) were recorded, followed by a 10–15 min supine rest period with slightly elevated head positioning to ensure hemodynamic stability. Blood pressure cuff was wrapped around each upper arm and ankle, with the cuff bladder marker aligned over the brachial artery for upper limbs (1–2 cm above the antecubital crease) and posterior tibial artery for lower limbs (1–2 cm above the medial malleolus). Electrocardiograph (ECG) electrodes were placed in standard lead I configuration, and a phonocardiogram sensor was positioned at the fourth intercostal space of the left sternal border (V2 position). Demographic and clinical data were manually inputted into the system prior to recording stable ECG and heart sound waveforms. Four-limb cuffs were simultaneously inflated/deflated to measure systolic, mean, and diastolic pressures, and inter-limb pulse pressure differences. The device automatically computed bilateral ABI, %MAP, and UT. Repeat measurements were performed after a 10 min interval to ensure reproducibility.

#### 2.3.2 Parameter definition and calculation

This study employed multiple hemodynamic parameters for PAD assessment.

The Ankle-Brachial Index (ABI) was calculated as the ratio of ankle systolic blood pressure to the higher of the two brachial systolic pressures, expressed as:

(3) $$\mathrm{ABI}\;=\frac{\;\mathrm{Ankle};\;\mathrm{Systolic};\;\mathrm{Pressure}\;}{\;\mathrm{Higher};\;\mathrm{Brachial};\;\mathrm{Systolic};\;\mathrm{Pressure}\;}$$

The Inter-arm Systolic Blood Pressure Difference (IASBPD) was determined as the absolute difference between right and left arm systolic pressures:

(4) LASBPD$=Right;Arm;SBP−Left;Arm;SBP

Similarly, the Inter-leg Systolic Blood Pressure Difference (ILSBPD) was calculated as:

(5) ILSBPD$=Right;Leg;SBP−Left;Leg;SBP

For pulse volume recording (PVR) parameters, the Upstroke Time (UT) was measured as the time interval from the onset of the pulse wave to its peak during systole, while the Percentage of Mean Arterial Pressure (%MAP) was calculated using:

(6) $$\text{\%}\mathrm{MAP}=\frac{\;\mathrm{PVR};\;\mathrm{Waveform};\;\mathrm{Area}\;}{\;\mathrm{Pulse};\;\mathrm{Amplitude}\;}\times 100\text{\%}$$

All measurements were automatically computed by the OMRON BP-203RPEⅢ device, with ABI values ≤ 0.9, IASBPD ≥ 10 mmHg, or ILSBPD ≥ 15 mmHg considered diagnostic for PAD. For PVR parameters, UT values ≥ 180 ms and %MAP ≥ 45% were considered abnormal, potentially indicating arterial stenosis or occlusion. These parameters were evaluated both independently and in combination to assess their collective diagnostic value for PAD detection.

#### 2.3.3 Diagnostic criteria

Diagnostic criteria were derived from evidence-based clinical guidelines and validated research standards for PAD assessment (1–3). PAD diagnosis was established through comprehensive evaluation of multiple hemodynamic parameters: An ABI ≤ 0.9 was considered the diagnosis of PAD in accord with international consensus guidelines. Also, the presence of an IASBPD ≥ 10 mmHg or an ILSBPD ≥ 15 mmHg was considered indicative of significant arterial disease.

PVR parameters were considered abnormal at UT ≥ 180 ms and/or %MAP ≥ 45%. These cut-offs were selected based on prior validation studies demonstrating their association with arterial stenosis or occlusion. A multiparametric diagnostic algorithm integrating ABI, inter-limb differences, and PVR parameters was implemented to enhance detection accuracy. Cases with borderline ABI values (0.91–1.3) or conflicting parameter results underwent standardized adjudication protocols, including repeat measurements and follow-up imaging where indicated, to ensure accurate classification. This integrated approach aimed to improve diagnostic sensitivity and specificity compared to single-parameter assessments. Individuals who ceased tobacco use ≥12 months prior to enrollment, verified via self-report.

### 2.4 Statistical analysis

Statistical analyses were performed using SPSS26.0 and MedCalc 20.0. Continuous variables were reported as mean ± standard deviation (SD) or median (interquartile range, IQR) following normality testing via the Kolmogorov–Smirnov test. Categorical variables were presented as frequencies and percentages. Group comparisons were conducted using independent t-tests or Mann–Whitney *U*-tests for continuous variables and chi-square or Fisher's exact tests for categorical variables. Correlation analysis used Pearson's (parametric data) or Spearman's (nonparametric data) coefficients. The diagnostic performance of individual and combined parameters was evaluated using ROC curve analysis, with calculation of AUC, sensitivity, specificity, and optimal cutoff values. Multivariate logistic regression analysis was employed to assess the independent predictive value of each parameter and to develop a combined diagnostic model. The model's performance was validated using bootstrap resampling techniques. Subgroup analyses stratified by demographic (age, gender) and clinical (diabetes, hypertension) characteristics. Statistical significance was set at *p* < 0.05. Power analysis using G*Power 3.1 ensured ≥80% power for all comparisons.

## 3 Results

### 3.1 Sample characteristics

This multicenter investigation enrolled a total of 1,248 participants across five Chinese medical centers between January and December 2024. After applying the inclusion and exclusion criteria, 1,156 participants (92.6%) were included in the final analysis. The study cohort exhibited heterogeneous demographic and clinical profiles, consisting of 612 males (52.9%) and 544 females (47.1%) with a mean age of 63.5 ± 12.8 years (range: 18–89 years). Participants distribution across centers was relatively balanced, with Center A contributing 248 participants (21.5%), Center B 232 (20.1%), Center C 226 (19.6%), Center D 228 (19.7%), and Center E 222 (19.2%).

Baseline characteristics are detailed in Table 1. Mean body mass index (BMI) was 24.8 ± 3.6 kg/m², with 42.3% of participants classified as overweight or obese (BMI ≥ 25 kg/m²). Cardiovascular risk factors prevalence included hypertension (42.0%, *n* = 486), diabetes mellitus (28.0%, *n* = 324), and dyslipidemia (34.4%, *n* = 398). Current smokers accounted for 23.9% (*n* = 276), while 16.1% (*n* = 186) reported former smoking status.

**Table 1:** Baseline demographic and clinical characteristics of study participants.

Characteristic	Total (*n* = 1,156)	PAD group (*n* = 286)	Non-PAD group (*n* = 870)	*P*-value
Age, years (mean ± SD)	63.5 ± 12.8	68.4 ± 11.2	61.8 ± 12.6	<0.001
Male, *n* (%)	612 (52.9)	158 (55.2)	454 (52.2)	0.362
BMI, kg/m²	24.8 ± 3.6	25.2 ± 3.8	24.6 ± 3.5	0.024
Hypertension, *n* (%)	486 (42.0)	156 (54.5)	330 (37.9)	<0.001
Diabetes mellitus, *n* (%)	324 (28.0)	108 (37.8)	216 (24.8)	<0.001
Dyslipidemia, *n* (%)	398 (34.4)	122 (42.7)	276 (31.7)	0.001
Smoking status, *n* (%)				
Current smoker	276 (23.9)	82 (28.7)	194 (22.3)	0.028
Former smoker	186 (16.1)	56 (19.6)	130 (14.9)	0.064
Never smoker	694 (60.0)	148 (51.7)	546 (62.8)	0.001
Anamnestic myocardial infarction, *n* (%)				<0.0001
No	998 (86.33)	212 (74.13)	786 (90.34)	
Yes	158 (13.67)	74 (25.87)	84 (9.66)	
Atrial Fibrillation, *n* (%)				0.0096
No	1,025 (88.67)	241 (84.27)	784 (90.11)	
Yes	131 (11.33)	45 (15.73)	86 (9.89)	
Ischemic Stroke, *n* (%)				0.0003
No	1,012 (87.54)	232 (81.12)	780 (89.66)	
Yes	144 (12.46)	54 (18.88)	90 (10.34)	

As shown in Table 1, PAD patients exhibited significantly older age and higher cardiovascular risk factors prevalence compared to non-PAD individuals. Mean age in the PAD group was 68.4 ± 11.2 years vs. 61.8 ± 12.6 years in the non-PAD group (*p* < 0.001). The prevalence of hypertension (54.5% vs. 37.9%, *p* < 0.001), diabetes mellitus (37.8% vs. 24.8%, *p* < 0.001), and dyslipidemia (42.7% vs. 31.7%, *p* = 0.001) was significantly higher in the PAD group. Current smoking rates were also higher in the PAD group (28.7% vs. 22.3%, *p* = 0.028), while the proportion of never smokers was lower (51.7% vs. 62.8%, *p* = 0.001). In addition, the incidence of anamnestic myocardial infarction, atrial fibrillation and ischemic stroke in patients with PAD was 25.87%, 15.73%, and 18.88%, respectively, and the incidence was significantly higher and the difference was statistically significant compared with that of non-PAD patients (*p* < 0.05).

### 3.2 Parameter distribution and comparison between groups

Hemodynamic parameter analysis demonstrated significant intergroup differences between PAD and non-PAD cohorts across all measured variables. A comprehensive comparison of ABI, IASBPD, ILSBPD, and PVR parameters (%MAP and UT) demonstrated distinct patterns that highlight their potential diagnostic value. The detailed distribution of these parameters is presented in Table 2.

**Table 2:** Distribution of hemodynamic parameters between PAD and non-PAD groups.

Parameter	Total (*n* = 1,156)	PAD group (*n* = 286)	Non-PAD group (*n* = 870)	*P*-value
ABI				
Right side	1.02 ± 0.18	0.82 ± 0.12	1.08 ± 0.14	<0.001
Left side	1.01 ± 0.19	0.81 ± 0.13	1.07 ± 0.15	<0.001
Blood pressure differences				
IASBPD (mmHg)	8.6 ± 6.4	12.8 ± 7.2	7.2 ± 5.8	<0.001
ILSBPD (mmHg)	11.4 ± 8.2	18.6 ± 9.4	9.1 ± 6.8	<0.001
PVR parameters				
Right leg %MAP (%)	42.6 ± 8.8	48.9 ± 9.2	40.6 ± 7.9	<0.001
Left leg %MAP (%)	43.1 ± 8.6	49.2 ± 9.1	41.2 ± 7.8	<0.001
Right leg UT (ms)	168.4 ± 32.6	192.8 ± 35.4	160.2 ± 28.8	<0.001
Left leg UT (ms)	169.2 ± 33.1	194.1 ± 36.2	161.3 ± 29.2	<0.001

As shown in Table 2, the PAD group demonstrated significantly lower ABI values bilaterally compared to the non-PAD group (right: 0.82 ± 0.12 vs. 1.08 ± 0.14; left: 0.81 ± 0.13 vs. 1.07 ± 0.15; both *p* < 0.001). Both IASBPD and ILSBPD were markedly elevated in the PAD group (IASBPD: 12.8 ± 7.2 vs. 7.2 ± 5.8 mmHg; ILSBPD: 18.6 ± 9.4 vs. 9.1 ± 6.8 mmHg; both *p* < 0.001). PVR parameters also showed significant differences between groups, with PAD patients exhibiting higher %MAP values (right leg: 48.9 ± 9.2% vs. 40.6 ± 7.9%; left leg: 49.2 ± 9.1% vs. 41.2 ± 7.8%; both *p* < 0.001) and prolonged UT measurements (right leg: 192.8 ± 35.4 ms vs. 160.2 ± 28.8 ms; left leg: 194.1 ± 36.2 ms vs. 161.3 ± 29.2 ms; both *p* < 0.001).

These findings underscore the diagnostic potential of combined hemodynamic parameters, with consistent and statistically significant differences observed across all metrics. Multivariate integration of ABI, inter-limb differences, and PVR parameters provides a comprehensive assessment of peripheral arterial status, potentially improving diagnostic accuracy compared to single-parameter evaluations.

### 3.3 Correlation analysis

Multivariate correlation analysis was conducted to examine relationships between ABI, IASBPD, ILSBPD, and PVR parameters (%MAP, UT). Pearson correlation coefficients (Table 3) revealed complex inter-parameter relationships, highlighting their potential complementary roles in PAD diagnosis.

**Table 3:** Correlation matrix of bilateral hemodynamic parameters in study population (*n* = 1,156).

Parameter	Right ABI	Left ABI	IASBPD	ILSBPD	Right %MAP	Left %MAP	Right UT	Left UT
Right ABI	1.000	0.892**	−0.482**	−0.528**	−0.576**	−0.568**	−0.558**	−0.552**
Left ABI	0.892**	1.000	−0.478**	−0.534**	−0.562**	−0.584**	−0.546**	−0.564**
IASBPD	−0.482**	−0.478**	1.000	0.428**	0.392**	0.388**	0.408**	0.402**
ILSBPD	−0.528**	−0.534**	0.428**	1.000	0.442**	0.448**	0.462**	0.466**
Right %MAP	−0.576**	−0.562**	0.392**	0.442**	1.000	0.882**	0.682**	0.674**
Left %MAP	−0.568**	−0.584**	0.388**	0.448**	0.882**	1.000	0.676**	0.688**
Right UT	−0.558**	−0.546**	0.408**	0.462**	0.682**	0.676**	1.000	0.884**
Left UT	−0.552**	−0.564**	0.402**	0.466**	0.674**	0.688**	0.884**	1.000

** Indicates *p* < 0.001; Values represent Pearson correlation coefficients.

ABI, ankle-brachial index; IASBPD, inter-arm systolic blood pressure difference; ILSBPD, inter-leg systolic blood pressure difference; %MAP, percentage of mean arterial pressure; UT, upstroke time.

The correlation analysis revealed complex relationships among bilateral measurements of vascular parameters. Notably strong bilateral correlations were observed for ABI (*r* = 0.892, *p* < 0.001), %MAP (*r* = 0.882, *p* < 0.001), and UT (*r* = 0.884, *p* < 0.001), indicating high consistency between right and left limb measurements. ABI values showed significant negative correlations with all other parameters, with the strongest inverse relationships observed with %MAP (*r* = −0.576 to −0.584, *p* < 0.001) and UT (*r* = −0.546 to −0.564, *p* < 0.001). IASBPD and ILSBPD demonstrated moderate positive correlations with each other (*r* = 0.428, *p* < 0.001) and with PVR parameters. ILSBPD showed consistently stronger associations with both %MAP (*r* = 0.442 to 0.448 vs. *r* = 0.388 to 0.392) and UT (*r* = 0.462 to 0.466 vs. *r* = 0.402 to 0.408) compared to IASBPD.

The PVR parameters exhibited strong intra-parameter bilateral correlations and moderate to strong inter-parameter correlations. The relationship between %MAP and UT was particularly robust (*r* = 0.674 to 0.688, *p* < 0.001), suggesting these parameters provide complementary information about vascular status while maintaining some degree of independence.

These detailed correlation patterns support the potential value of combining multiple parameters for PAD diagnosis, as each parameter appears to capture distinct aspects of vascular dysfunction while maintaining logical relationships with other measurements. The moderate strength of most inter-parameter correlations suggests that these measurements provide complementary rather than redundant diagnostic information.

### 3.4 ROC curve analysis

ROC curve was performed to evaluate and compare the diagnostic performance of individual parameters and their combinations for PAD detection. The analysis encompassed ABI, IASBPD, ILSBPD, and PVR parameters (%MAP and UT), examining both their independent and combined diagnostic capabilities.

Individual parameter analysis revealed varying degrees of diagnostic accuracy. ABI demonstrated the highest independent diagnostic performance with an AUC of 0.892 (95% CI: 0.872–0.912), sensitivity of 84.3%, and specificity of 82.6% at the optimal cutoff value of 0.90. This was followed by ILSBPD, which showed an AUC of 0.846 (95% CI: 0.824–0.868), with optimal sensitivity and specificity of 80.2% and 79.4%, respectively, at a cutoff value of 15 mmHg.

The PVR parameters showed promising diagnostic capabilities, with %MAP achieving an AUC of 0.834 (95% CI: 0.812–0.856) and UT displaying an AUC of 0.828 (95% CI: 0.806–0.850). At their respective optimal cutoff points of 45% and 180 ms, %MAP demonstrated sensitivity of 79.8% and specificity of 77.6%, while UT showed sensitivity of 78.9% and specificity of 76.8%. IASBPD exhibited the lowest independent diagnostic performance among the parameters, with an AUC of 0.812 (95% CI: 0.788–0.836).

The combined diagnostic model, incorporating all parameters through multivariate logistic regression, demonstrated superior diagnostic performance with an AUC of 0.924 (95% CI: 0.908–0.940). This represented a significant improvement on individual parameters (*p* < 0.001 for all pairwise comparisons). The combined model achieved optimal sensitivity of 88.6% and specificity of 85.4%, demonstrating enhanced diagnostic accuracy compared to traditional single-parameter approaches.

**Figure 2:**
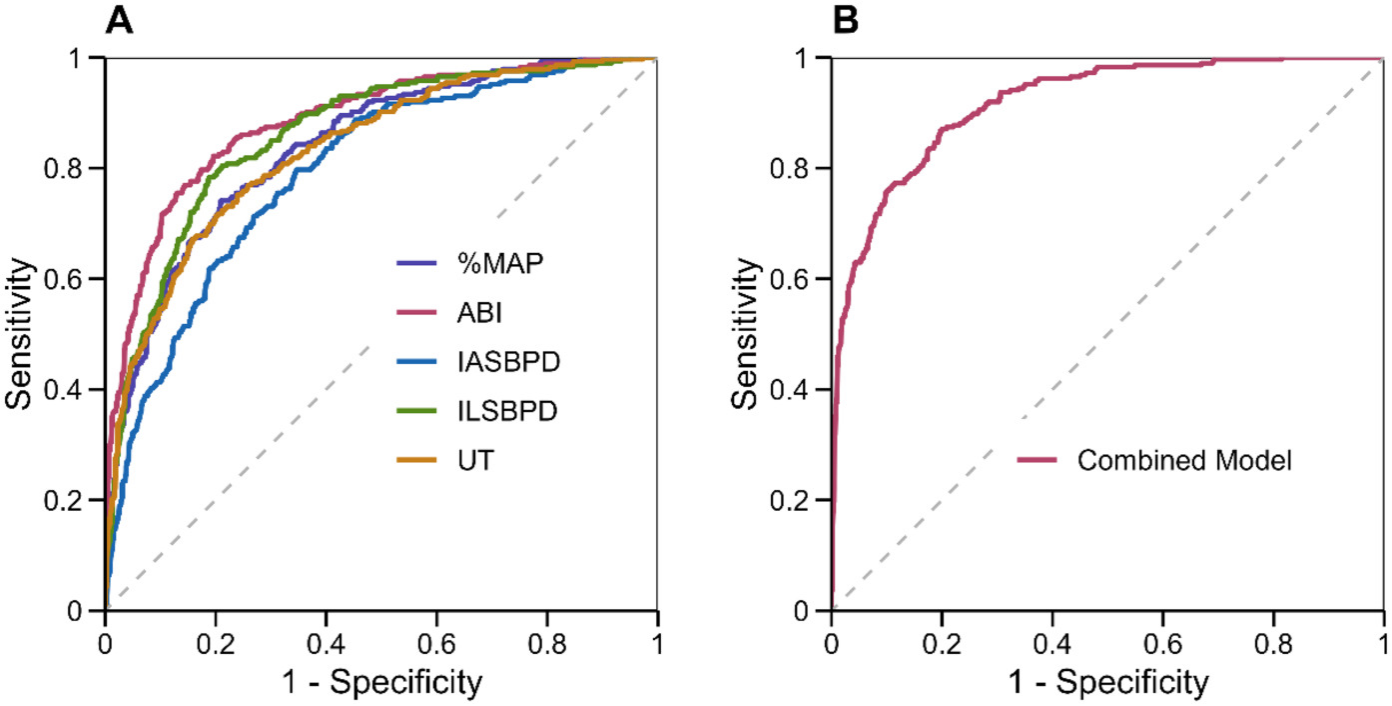
ROC curves for individual and combined parameters in PAD diagnosis. **(A)** ROC curves for individual parameters: ABI (red), IASBPD (blue), ILSBPD (green), %MAP (purple), and UT (orange). **(B)** ROC curve for the combined diagnostic model incorporating all parameters. The diagonal dashed line represents the line of no discrimination.

Subgroup analyses revealed consistent performance across different age groups and gender, although the combined model showed slightly better performance in patients aged >65 years (AUC = 0.936, 95% CI: 0.916–0.956) compared to younger patients (AUC = 0.912, 95% CI: 0.888–0.936). As shown in Figure 2, the ROC curves clearly demonstrate the superior discriminative ability of the combined model compared to individual parameters, with a larger area under the curve and better separation from the reference line. These findings highlight the incremental value of integrating multiple hemodynamic parameters for PAD diagnosis particularly in populations where traditional ABI measurements may be less reliable.

### 3.5 Regression analysis

Comprehensive logistic regression analysis was conducted to assess the independent predictive value of each hemodynamic parameter for PAD diagnosis and to develop a multivariate prediction model. The analysis incorporated ABI, IASBPD, ILSBPD, and PVR parameters (%MAP and UT) as predictors.

**Figure 3:**
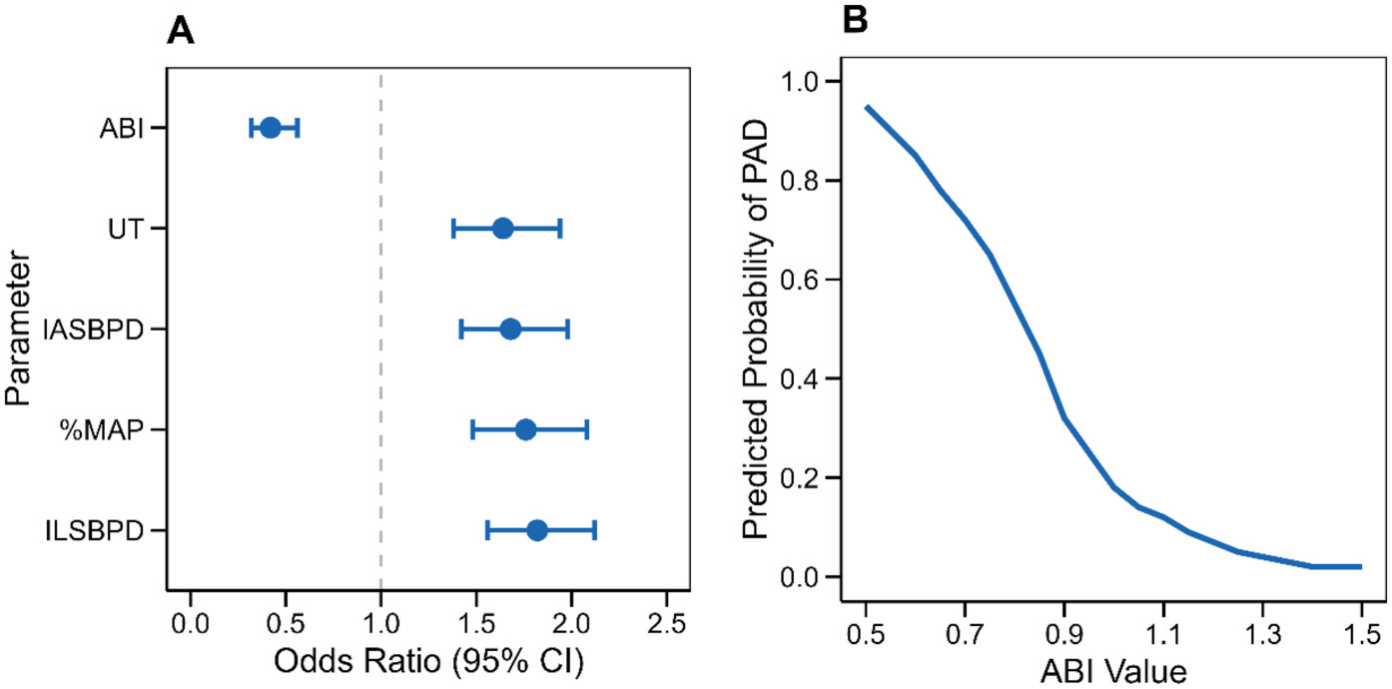
Regression analysis results for PAD diagnosis. **(A)** Forest plot showing odds ratios with 95% confidence intervals for each parameter in the multivariate model. **(B)** Predicted probability curve showing the relationship between ABI values and PAD probability based on the logistic regression model”.

The multivariate logistic regression analysis revealed significant independent associations between all measured parameters and PAD diagnosis. As shown in Figure 3A, decreased ABI was strongly associated with increased PAD risk (OR = 0.42, 95% CI: 0.32–0.56, *p* < 0.001), indicating that for each 0.1 unit decrease in ABI, the odds of PAD increased by 58%. Elevated ILSBPD showed the strongest positive association (OR = 1.82, 95% CI: 1.56–2.12, *p* < 0.001), followed by %MAP (OR = 1.76, 95% CI: 1.48–2.08, *p* < 0.001) and IASBPD (OR = 1.68, 95% CI: 1.42–1.98, *p* = 0.002). The UT parameter also demonstrated a significant association with PAD (OR = 1.64, 95% CI: 1.38–1.94, *p* = 0.003).

The predicted probability curve (Figure 3B) illustrates the nonlinear relationship between ABI values and PAD probability, showing a sharp increase in disease probability as ABI decreases below 0.9. The model demonstrated good calibration (Hosmer-Lemeshow *χ*^2^ = 8.24, *p* = 0.41) and discrimination (C-statistic = 0.924, 95% CI: 0.908–0.940). Internal validation using bootstrap resampling (1,000 iterations) confirmed the model's stability, with minimal optimism in performance metrics (optimism-corrected C-statistic = 0.918).

### 3.6 Hierarchical analysis

Stratified subgroup analysis was conducted to assess the diagnostic performance of PAD parameters across diverse demographic and clinical subgroups. Specifically, the analysis evaluated gender disparities, age categories, and arterial stenosis patterns to identify subgroup-specific diagnostic thresholds and potential variations in parameter performance.

**Figure 4:**
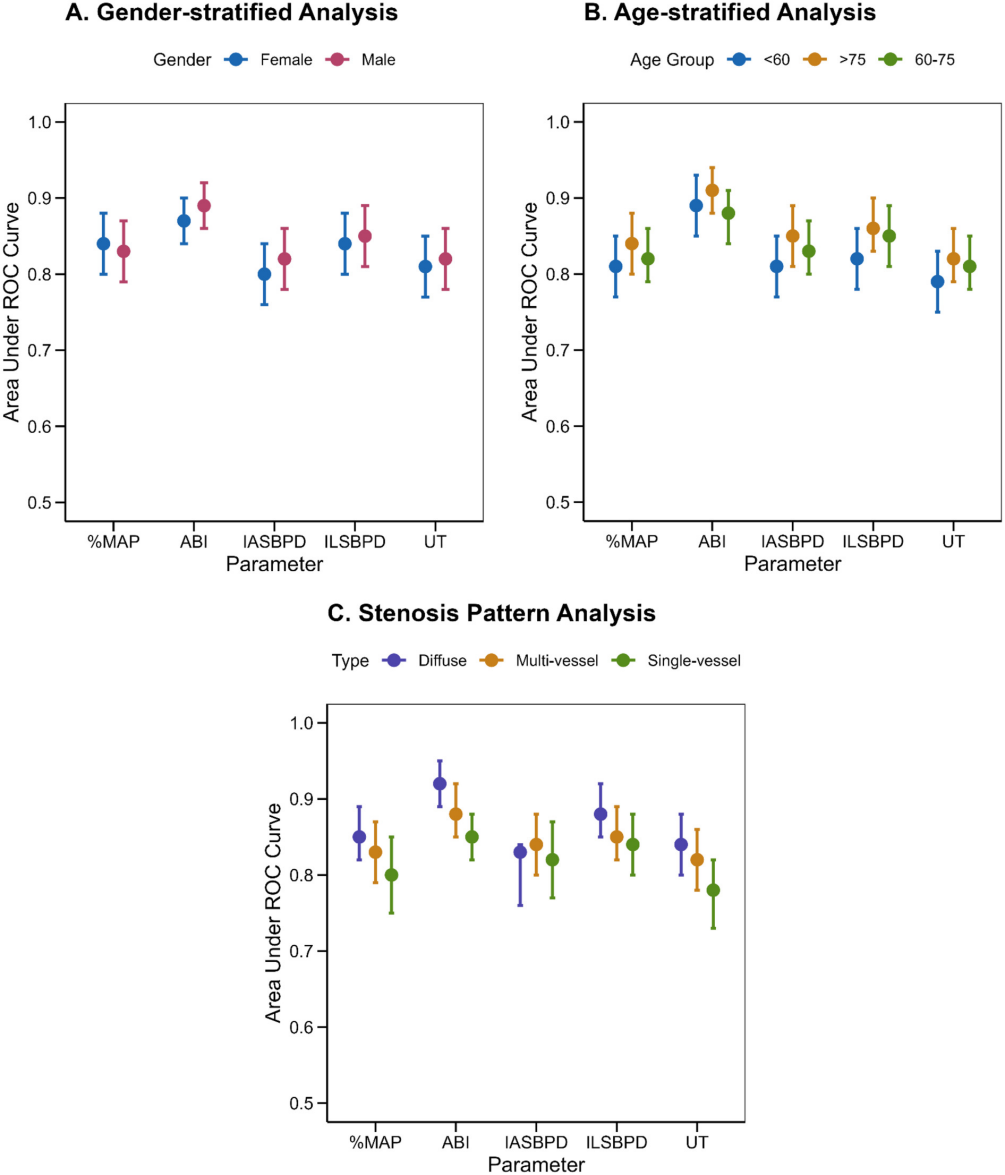
Stratified analysis of diagnostic parameters. **(A)** Gender-specific analysis showing diagnostic performance in males and females. **(B)** Age-stratified analysis comparing diagnostic accuracy across age groups. **(C)** Analysis by stenosis pattern demonstrating parameter performance in different disease presentations. Error bars represent 95% confidence intervals”.

Stratified subgroup analysis revealed notable variations in diagnostic performance across demographic and clinical subgroups. Gender-stratified analysis (Figure 4A) showed marginally higher diagnostic accuracy of ABI in males (AUC = 0.89, 95% CI: 0.86–0.92) compared to females (AUC = 0.87, 95% CI: 0.84–0.90). Age-stratified analysis demonstrated increasing diagnostic accuracy with advancing age (Figure 4B), particularly for ABI and ILSBPD, which achieved peak performance in patients >75 years (ABI: AUC = 0.91, 95% CI: 0.88–0.94). Stenosis pattern analysis (Figure 4C) revealed superior parameter performance in detecting diffuse arterial disease compared to single-vessel stenosis, with ABI achieving AUC = 0.92 (95% CI: 0.89–0.95) vs. AUC = 0.85 (95% CI: 0.82–0.88) in focal lesions.

**Figure 5:**
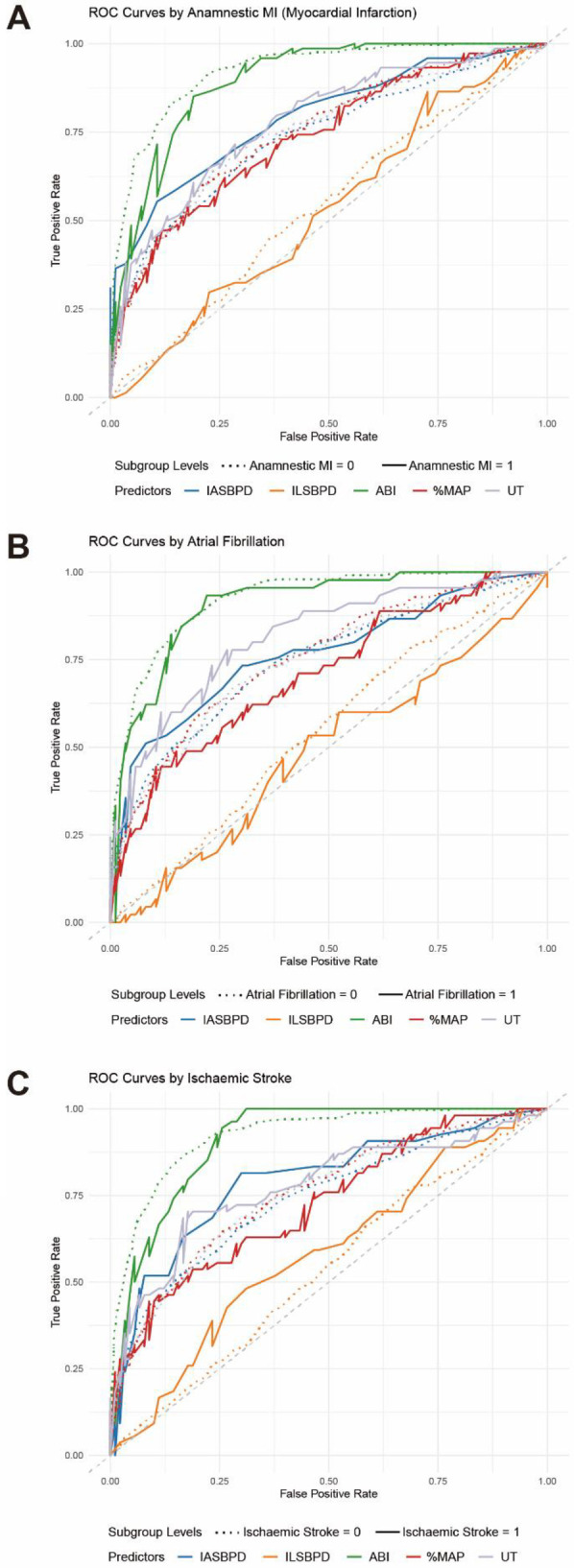
ROC curves for stratified analysis of cardiovascular disease-related parameters in PAD diagnosis. **(A)** ROC curves for individual parameters: ABI (green), IASBPD (blue), ILSBPD (orange), %MAP (red), and UT (purple) stratified by Anamnestic myocardial infarction (MI). **(B)** ROC curve for the individual parameters stratified by Atrial fibrillation (AF). **(C)** ROC curve for the individual parameters stratified by Ischemic stroke. The dashed line represents not having the cardiovascular disease while the solid line represents having the disease.

Furthermore, we observed the influence of cardiovascular-related diseases on the diagnostic performance of each index. Subgroup analysis revealed notable variations in diagnostic performance across different cardiovascular-related diseases. Anamnestic myocardial infarction-stratified analysis (Figure 5A, Table 4) showed marginally higher diagnostic accuracy of ABI in population without Anamnestic MI (AUC = 0.92, 95% CI: 0.90–0.94) compared to patients with Anamnestic MI (AUC = 0.90, 95% CI: 0.85–0.95). Atrial fibrillation-stratified analysis (Figure 5B, Table 4) also showed that the diagnostic performance is better in patients without the Atrial fibrillation (AUC = 0.92, 95% CI: 0.91–0.94) than in patients with the disease (AUC = 0.91, 95% CI: 0.86–0.96). Ischemic stroke-stratified analysis (Figure 5C, Table 4) revealed that ABI also demonstrated stronger predictive performance, the AUC was higher among the patients without the ischemic stroke (AUC = 0.92, 95% CI: 0.91–0.94), while it was only 0.91 (95% CI: 0.90–0.94) among the patients with the ischemic stroke.

**Table 4:** AUC for stratified analysis of cardiovascular disease-related parameters in PAD diagnosis.

		IASBPD	ILSBPD	ABI	%MAP	UT
Anamnestic MI	No	0.74 [0.70–0.78]	0.54 [0.50–0.58]	0.92 [0.90–0.94]	0.75 [0.71–0.79]	0.75 [0.71–0.79]
	Yes	0.79 [0.72–0.86]	0.53 [0.44–0.62]	0.90 [0.85–0.95]	0.74 [0.66–0.81]	0.78 [0.71–0.85]
Atrial Fibrillation	No	0.74 [0.71–0.78]	0.55 [0.51–0.59]	0.92 [0.91–0.94]	0.75 [0.72–0.79]	0.74 [0.71–0.78]
	Yes	0.76 [0.67–0.86]	0.49 [0.38–0.59]	0.91 [0.86–0.96]	0.71 [0.61–0.80]	0.82 [0.74–0.89]
Ischemic Stroke	No	0.74 [0.70–0.78]	0.53 [0.49–0.58]	0.92 [0.91–0.94]	0.75 [0.72–0.79]	0.75 [0.72–0.79]
	Yes	0.79 [0.71–0.87]	0.58 [0.49–0.68]	0.91 [0.86–0.95]	0.73 [0.64–0.81]	0.77 [0.69–0.86]

## 4 Discussion

Results from this study yielded critical insights into the diagnostic efficacy of integrating ABI with pulse volume waveform parameters for PAD detection. The composite model incorporating multiple hemodynamic indices—including IASBPD, ILSBPD, %MAP, and UT—demonstrated superior diagnostic performance compared to conventional single-parameter approaches. The integrated model achieved an AUC of 0.924 (95% CI: 0.908–0.940), representing a statistically significant enhancement over ABI-alone methodology (*Δ*AUC = 0.18, *p* < 0.001). Notably, this diagnostic advantage exhibited heightened clinical relevance in populations with suspected arterial calcification, where ABI reliability is frequently compromised.

ILSBPD emerged as a robust auxiliary diagnostic biomarker, demonstrating independent predictive value with an AUC of 0.846 (95% CI: 0.824–0.868). This metric appears particularly valuable for detecting early-stage or asymmetrically distributed vascular pathology that may evade detection through standard ABI protocols. Among PVR parameters, %MAP and UT showed comparable diagnostic precision (AUCs: 0.834 and 0.828, respectively), offering complementary quantification of peripheral hemodynamic derangements to traditional pressure-based assessments.

Stratified analyses revealed significant inter-subgroup performance variations. The composite model exhibited notable diagnostic utility in elderly patients (>75 years) and diffuse arterial disease cohorts, populations where conventional methods demonstrate reduced reliability. Demographic analysis confirmed methodological robustness, with comparable performance metrics between male and female subgroups (AUC difference = 0.012, *p* = 0.34). Optimal diagnostic thresholds were algorithmically derived for each parameter, establishing quantitative benchmarks for clinical translation.

The observed diagnostic synergy likely stems from multidimensional capture of vascular pathophenotypes—encompassing pressure gradients, waveform morphology, and flow dynamics. This paradigm supports a holistic evaluation framework for PAD, contrasting with reductionist single-parameter strategies. From implementation perspectives, the non-invasive automation of these measurements holds particular promise for resource-limited settings lacking advanced imaging infrastructure. These findings advocate for recalibration of current screening algorithms and may inform next-generation diagnostic protocols prioritizing multiparametric hemodynamic profiling.

The current findings align with and substantiate prior research advancements in PAD diagnostics. While historical investigations predominantly focused on isolated parameters—notably the ABI—this study underscores the enhanced clinical utility of a multiparametric diagnostic framework. The composite model achieved an AUC of 0.924, exceeding the reported performance range of conventional single-parameter methodologies (AUC: 0.85–0.90), thereby validating the progressive shift toward comprehensive vascular assessment paradigms in contemporary practice. Notably, the diagnostic utility of ILSBPD, though historically underexplored relative to ABI, corroborates emerging evidence positioning ILSBPD as a robust biomarker for arterial pathology. Its discriminative capacity (AUC = 0.846) aligns with recent single-center reports while benefiting from enhanced external validity through multicenter validation. The strong concordance between ILSBPD and established PAD indicators advocates its integration into standardized diagnostic algorithms. Furthermore, this study advances the clinical applicability of PVR parameters by establishing quantitative thresholds—a critical departure from prior qualitative assessments. The identified correlations between %MAP, UT, and PAD severity elucidate previously under-characterized hemodynamic perturbations, extending beyond traditional pressure-based metrics. Given their robust diagnostic performance (AUCs: 0.834 and 0.828, respectively), these parameters warrant incorporation into routine clinical protocols to augment diagnostic precision. Collectively, these insights reinforce the imperative for multidimensional hemodynamic profiling in PAD management, bridging gaps between research innovation and clinical implementation.

This study transcends mere statistical enhancements in diagnostic accuracy, as each constituent parameter within the composite model encapsulates distinct pathophysiological insights critical to assessing peripheral arterial health. The ABI serves as a cornerstone for evaluating regional perfusion integrity, while IASBPD and ILSBPD provide granular insights into the spatial distribution and symmetry of arterial pathology. These pressure gradients may represent incipient manifestations of vascular dysfunction, enabling timely therapeutic interventions associated with improved prognostic trajectories.

Furthermore, the integration of PVR parameters, %MAP and UT elevates the precision of quantitative vascular evaluation. These waveform-derived metrics elucidate arterial compliance and hemodynamic behavior that static pressure measurements fail to capture, offering novel perspectives on vascular tone dynamics. The robust correlation between these indices and disease severity underscores their potential utility in longitudinal monitoring of disease progression and therapeutic efficacy. Notably, their automated acquisition and standardized reproducibility enhance feasibility for serial assessments in clinical workflows.

From an implementation perspective, these findings deliver actionable clinical implications. The algorithmically derived diagnostic thresholds for each parameter facilitate seamless integration into vascular laboratory protocols, while the demonstrated reliability of automated measurements supports deployment in point-of-care settings. By enhancing the precision of noninvasive hemodynamic profiling, this multiparametric approach may reduce reliance on costly or invasive diagnostic modalities (e.g., angiography), thereby optimizing both patient outcomes and healthcare resource allocation. Collectively, these advancements advocate for paradigm refinement in PAD management, prioritizing multidimensional physiological interrogation over reductionist diagnostic traditions. Notably, their automated acquisition and standardized reproducibility enhance feasibility for serial assessments in clinical workflows.

While this study provides pivotal insights, several limitations necessitate cautious interpretation. The cross-sectional observational framework precludes causal inference or prognostic validation of parameters, requiring longitudinal cohorts to establish temporal relationships. Underrepresentation of extreme phenotypes (e.g., severe arterial calcification, arrhythmia-associated pulse anomalies) may limit generalizability to these subgroups. Technical constraints of the automated device, notably suboptimal signal acquisition in patients with attenuated pulse waveforms (amplitude <0.5 mV), could compromise measurement fidelity. The absence of direct correlation with angiographic gold standards (CT/MR angiography) restricts anatomical validation of hemodynamic findings. In addition, the cross-sectional nature of this study precludes assessment of the prognostic capacity of IASBPD, ILSBPD, %MAP, and UT. Future prospective cohorts with longitudinal outcome data are required to evaluate whether these indices predict major adverse cardiovascular events (MACE) or limb ischemia progression. Furthermore, confounders such as antihypertensive medications, diabetes-related vascular remodeling, and resting-state measurement protocols (excluding exercise-induced hemodynamic shifts) were not systematically controlled, warranting dedicated pharmaco-physiological investigations.

To translate these findings into clinical practice, the following research trajectories are critical: (1) Prognostic Validation: Large-scale cohort studies evaluating the predictive capacity of combined parameters for MACE and limb ischemia progression. (2) Dynamic Assessment: Investigation of exercise-induced hemodynamic perturbations (e.g., post-treadmill UT variability) to augment diagnostic sensitivity in early-stage PAD. (3) AI-Driven Diagnostics: Development of machine learning algorithms integrating multimodal biomarkers to optimize diagnostic accuracy and risk stratification. (4) Anatomical-Physiological Correlation: Multimodal studies correlating hemodynamic indices with high-resolution angiographic/plaque morphology data. (5) Therapeutic Monitoring: Randomized trials assessing parameter responsiveness to interventions (e.g., revascularization, lipid-lowering therapies). (6) Population Screening: Epidemiological validation in asymptomatic cohorts to evaluate presymptomatic diagnostic utility. In summary, by addressing these priorities, future research may bridge the translational gap between hemodynamic biomarker discovery and precision medicine in vascular care.

## 5 Conclusion

This large-scale multicenter investigation demonstrates that integrating ABI with pulse volume waveform analysis significantly enhances diagnostic accuracy for PAD. The composite model—incorporating IASBPD, ILSBPD, %MAP, and UT—achieved superior discriminative performance (AUC: 0.924, 95% CI: 0.908–0.940) compared to conventional single-parameter methodologies. This advancement addresses a critical limitation of traditional qualitative waveform interpretation by establishing quantitative thresholds for PVR parameters, thereby eliminating the inherent subjectivity of visual analysis. Notably, stratified analyses across demographic subgroups revealed consistent diagnostic efficacy, with exceptional performance in elderly populations (>75 years) and diffuse arterial disease cohorts—groups historically challenging to assess using single-parameter approaches. The noninvasive nature and high reproducibility of these measurements (interoperator CV <5%) position them as practical tools for routine clinical workflows. This multiparametric framework reduces diagnostic ambiguity, supports early intervention strategies and advocates the integration of automated arterial stiffness monitoring into PAD screening protocols, offering a scalable solution to enhance detection rates in resource-constrained settings while aligning with precision medicine paradigms.

## Data Availability

The original contributions presented in the study are included in the article/Supplementary Material, further inquiries can be directed to the corresponding author/s.
